# Learning from Sensory and Reward Prediction Errors during Motor Adaptation

**DOI:** 10.1371/journal.pcbi.1002012

**Published:** 2011-03-10

**Authors:** Jun Izawa, Reza Shadmehr

**Affiliations:** 1 Department of Biomedical Engineering, Johns Hopkins School of Medicine, Baltimore, Maryland, United States of America; 2 Department of Human Media Systems, The University of Electro-Communication, Chofu, Tokyo, Japan; Northwestern University, United States of America

## Abstract

Voluntary motor commands produce two kinds of consequences. Initially, a sensory consequence is observed in terms of activity in our primary sensory organs (e.g., vision, proprioception). Subsequently, the brain evaluates the sensory feedback and produces a subjective measure of utility or usefulness of the motor commands (e.g., reward). As a result, comparisons between predicted and observed consequences of motor commands produce two forms of prediction error. How do these errors contribute to changes in motor commands? Here, we considered a reach adaptation protocol and found that when high quality sensory feedback was available, adaptation of motor commands was driven almost exclusively by sensory prediction errors. This form of learning had a distinct signature: as motor commands adapted, the subjects altered their predictions regarding sensory consequences of motor commands, and generalized this learning broadly to neighboring motor commands. In contrast, as the quality of the sensory feedback degraded, adaptation of motor commands became more dependent on reward prediction errors. Reward prediction errors produced comparable changes in the motor commands, but produced no change in the predicted sensory consequences of motor commands, and generalized only locally. Because we found that there was a within subject correlation between generalization patterns and sensory remapping, it is plausible that during adaptation an individual's relative reliance on sensory vs. reward prediction errors could be inferred. We suggest that while motor commands change because of sensory and reward prediction errors, only sensory prediction errors produce a change in the neural system that predicts sensory consequences of motor commands.

## Author Summary

It is thought that motor adaptation relies on sensory prediction errors to form an estimate of the perturbation. Here, we present evidence that motor adaptation can be driven by both sensory and reward prediction errors. We found that learning from sensory prediction error altered the predicted consequences of motor commands, leaving behind a sensory remapping, whereas learning from reward prediction error produced comparable change in motor commands, but did not produce a sensory remapping. It is possible that the neural basis of learning from sensory and reward prediction errors are distinct because they produce different generalization patterns.

## Introduction

Our motor commands generally produce two kinds of consequences: a sensory consequence in terms of activity in our primary sensory organs (e.g., vision, proprioception), and a rewarding consequence in terms of forming a subjective measure of utility or usefulness of these sensations (e.g., release of dopamine). For example, while dancing, the motor commands that move our body produce proprioceptive feedback, while internal evaluation of that feedback indicates a pleasurable experience. These two consequences of the motor command form the basis for two kinds of prediction error: a sensory prediction error, and a reward prediction error. In principle, learning from sensory prediction error should alter an internal model that predicts the sensory consequences of motor commands, i.e., a forward model [1], [2]. In contrast, learning from reward prediction error should alter the valuation of the sensory states that are the consequence of those motor commands, i.e., a value function. Motor adaptation studies often focus on learning from sensory prediction error [1], [2], [3], [4], [5], [6], [7], despite the fact that people are also rewarded for each movement. Similarly, studies that focus on learning from reward prediction error (e.g., decision making tasks) often do not consider potential sensory prediction errors [8], [9], [10]. It seems rational that most learning would rely on both kinds of error. Here, we focus on a simple motor adaptation task and consider a mathematical framework in which both reward and sensory prediction errors could contribute to the trial-to-trial change in the motor commands. We attempt to ask whether learning from these two distinct signals can be behaviorally dissociated.

Our idea is that while motor commands might change because of sensory or reward prediction errors, only in the former case would there also be a change in the map that predicts the sensory consequences of the motor command. We focus on a well studied motor adaptation protocol: reaching in the context of visuomotor perturbations. While there have been numerous models of motor adaptation [4], [5], [11], [12], [13], [14], to our knowledge all current models assume that the process of motor adaptation is driven by sensory prediction errors. Our objective is to test the hypothesis that during motor adaptation, learning from sensory prediction errors leaves a behavioral signature that is distinct from learning from reward prediction errors.

## Results

**Figure 1:**
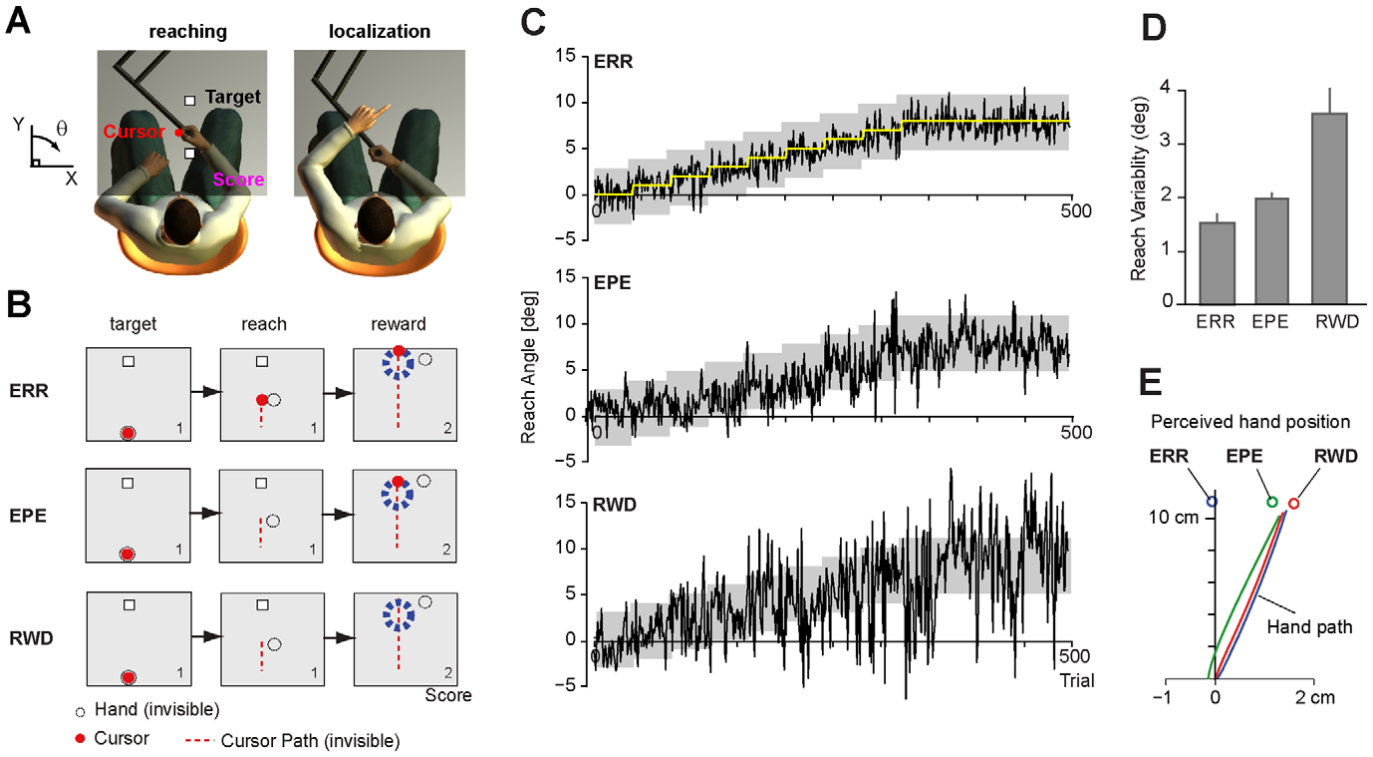
Experimental setup. (**A**) In the reaching task, subjects held a handle of a robotic arm and made ‘shooting’ movements to move a cursor through a target at 10 cm. The arm was covered by a screen. During adaptation, the cursor-hand relationship was perturbed so that the cursor position was rotated around the center at the start position. The coordinate system is drawn on the left side of the robot (invisible for subject) where the clockwise rotation around the start is positive. The cumulative score of each block was provided to the subject. In the localization task, subjects pointed with their left hand over the screen to the remembered location of their right hand as it crossed the (unseen) target area in the previous trial. In the localization task, the start box was not visible. (**B**) Experimental paradigms. In ERR, full visual feedback about the cursor position was provided as well as the animation and the sound indicating target explosion regarding success or failure of the task. In EPE, while the cursor was unseen during the shooting movement, it was presented for 200 ms as the hand crossed an imaginary circle with the radius equal to the target, providing endpoint error with respect to the target. The reward signal was also provided as in the ERR condition. In RWD, no visual feedback about the cursor was provided. All information that subjects were able to use was the success or failure of the task. (**C**) Reach angles of three representative subjects during the adaptation phase. The yellow line in the ERR group is the ideal reach angle, which shifted gradually up to 8 degrees by the visual rotation. The gray area indicates the reward region, which shifted with the same schedule in the three groups. (**D**) Reach variability in the final 100 trials for each group. There are the significant differences between ERR and EPE (t-test, p<0.003) as well as between EPE and RWD (t-test, p<0.001). (**E**) Results of the localization task for the three subjects. The reach trajectory is plotted for the POST condition. Red line is for the RWD subject, blue line is for the ERR subject, and green line is for the EPE subject. The circle around the reach trajectory is the averaged pointing location in the localization trial.

Consider a typical adaptation task in which the learner experiences a perturbation. The limb is covered by a screen to prevent direct observation of the hand, and a cursor that represents hand position undergoes a kinematic rotation so that when the hand moves straight ahead, the cursor moves slightly to the left (Fig. 1A). Reward is provided if the cursor passes through the target area. In this reach adaptation task there are two kinds of error: the difference between the expected and observed visual feedback of the hand (i.e. visual cursor), and the difference between the expected and observed success of the reach. Our hypothesis is that learning mechanisms engaged by the two types of error may be behaviorally dissociable.

To examine this hypothesis, we recruited two groups of subjects in Experiment 1. One group (RWD) was provided only with information regarding whether they succeeded or failed at each trial (reward r = 1 or 0), indicated by explosion of the target, and received no other visual feedback regarding their movement (Fig. 1B). Another group was provided with full visual feedback of the cursor as well as the reward so that they were able to use both potential error signals (ERR). We asked two questions: 1) In the ERR paradigm in which sensory consequences of motor commands were available, would adaptation of the motor commands accompany a change in the motor-sensory map (i.e., a change in the perceived sensory consequences of motor commands), and 2) in the RWD paradigm in which sensory consequences of motor commands were unavailable, would adaptation of the motor commands take place but without a change in the motor-sensory map.

Fig. 1C shows data from representative subjects in the ERR and RWD paradigms. In this figure, the yellow line in the ERR group is the ideal reach angle (shifts gradually up to 8°). The gray area indicates the region that provided reward, which shifts with the same schedule in both groups. The subjects were provided with different kinds of error feedback, but updated their motor commands by roughly the same amount (group data, mean change in reach direction, 7.49° for RWD and 7.63° for ERR, not significantly different from each other p>0.8, t-test). The total amount of adaptation of the two groups was comparable. However, the variability of reach angles was larger for the RWD subject (Fig. 1C), and this was consistent across the entire group (Fig. 1D).

Before and after this adaptation task (PRE and POST adaptation), we measured how subjects predicted the sensory consequences of their motor commands. In this localization part of the task, after subjects completed a reach with their right hand, their hand was returned to the center location, and they were then asked to estimate the location of their right hand in the previous trial by pointing with their left hand over the screen (Fig. 1A). During the localization neither the cursor nor the target was projected. The localization data for representative subjects are shown in Fig. 1E. As a consequence of adaptation, the subject in the ERR group had a sensory remapping in which she estimated her hand to be to the left of its actual position. In contrast, the subject in the RWD group had little or no sensory remapping, suggesting that the changes in the motor commands did not accompany a change in the motor-sensory map.

**Figure 2:**
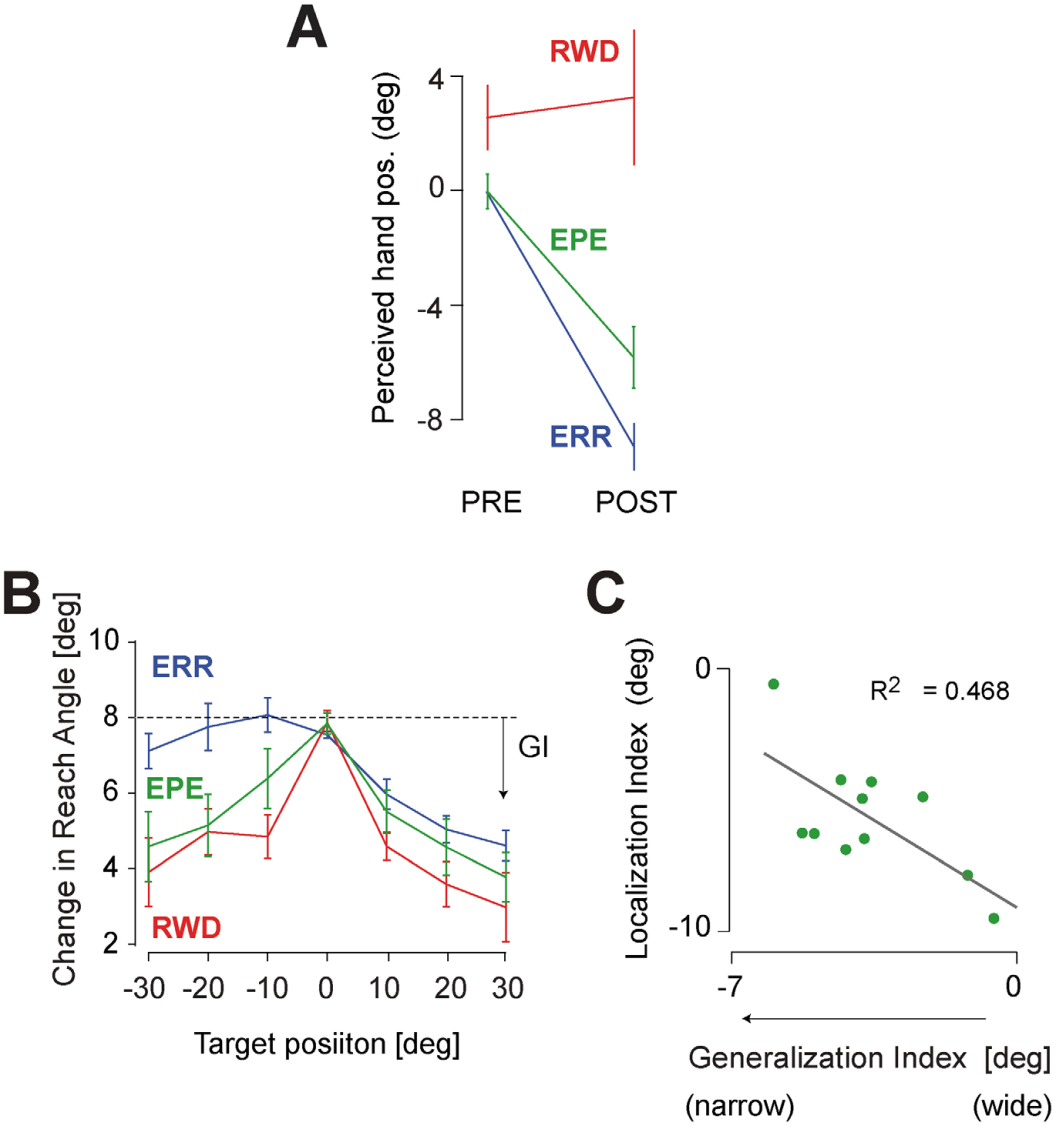
The sensory remapping and the generalization function. (**A**) The average estimated localization of hand position in PRE and POST conditions. Error bars are SEM. (**B**) Generalization of adaptation from the learned target direction (at 0°) to neighboring target directions. (**C**) Illusion index (change in estimated location of the hand from PRE to POST adaptation), as a function of generalization index in subjects in EPE condition. Each dot indicates individual subject's data. There are significant negative correlation in these two indices (R = −0.68, p = 0.02).

Fig. 2A shows the group data for the localization task. We compared the change in the estimate of hand position from the PRE to the POST adaptation condition and found that the subjects in the ERR group estimated their hand position to have changed by 8.8°+/−0.6° to the left of actual position. In contrast, in the POST condition of the RWD group, the subjects had no significant change in their sensory estimates (there was a significant difference between PRE and POST in the ERR group p<0.0001, whereas the difference in the RWD group was not significant p = 0.8).

If the sensory and reward prediction errors engage learning in distinct neural structures, then adaptation might result in distinct generalization patterns [15], [16], [17], [18]. To test this idea, we recruited subjects for Experiment 2 and quantified the patterns of generalization that accompanied adaptation. In the adaptation session, the target was projected at 0° (straight ahead). In the pre and post adaptation periods the target appeared randomly at various angular displacements (−30 to 30 deg). For these generalization targets, we provided neither the cursor nor reward information. Fig. 2B plots the average reach angle across subjects for each target direction. We found that the RWD group had a narrower generalization function than the ERR group (ANOVA, F(1,126) = 9.632, p = 0.005). In summary, in the RWD condition the learning that produced changes in the motor commands accompanied a narrow generalization function and no change in the map that predicted the sensory consequences of motor commands. In contrast, in the ERR paradigm the learning that produced changes in the motor commands accompanied a broad generalization function and a significant change in the perceived sensory consequences of motor commands.

In the RWD paradigm the binary feedback signal carried much less information than the continuous sensory error signal available in the ERR paradigm. This may have forced the subjects to adopt a completely new strategy, making the learning that we see in the RWD paradigm irrelevant for a typical adaptation paradigm. In Experiment 3 we considered a paradigm (EPE) in which the visual cursor was available only at the endpoint of the movement and was otherwise invisible during the reach. In this new experiment we measured the localization change (as in Exp. 1) and the generalization (as in Exp. 2), attempting to test the results of experiments 1 and 2 in the same population.

Fig. 1C shows the reach angles of a representative subject in the EPE group. The adaptation in the EPE group was comparable with the ERR group (mean change in reach direction, t-test, p = 0.64), i.e., the motor commands in the three groups adapted by approximately the same amount. Interestingly, in the localization task the subject in the EPE group had a sensory illusion that was in between the ERR and RWD groups (Fig. 1E). In the group data in the POST adaptation condition, the strength of the localization illusion in the EPE group was weaker than in the ERR group (t-test, p<0.007), but stronger than the RWD group (t-test, p<0.006) (Fig. 2A). The generalization of the EPE group appeared to be in between ERR and RWD (we did not see a significant difference from either ERR or RWD, Fig. 2B). In Experiment 2 we had found that learning from reward produced a narrow generalization, while in Experiment 1 we had found that learning from error produced a motor-sensory remapping. In Experiment 3 we had the means to test a crucial prediction: across subjects, individuals who relied more on reward (narrow generalization) should show a smaller motor-sensory remapping. Indeed, we found a significant correlation between the amount of generalization and the localization illusion across subjects (Fig. 2C). That is, it appeared that when a subject had a larger sensory illusion (suggesting that learning was driven more by sensory prediction errors), they also had a wider generalization.

**Figure 3:**
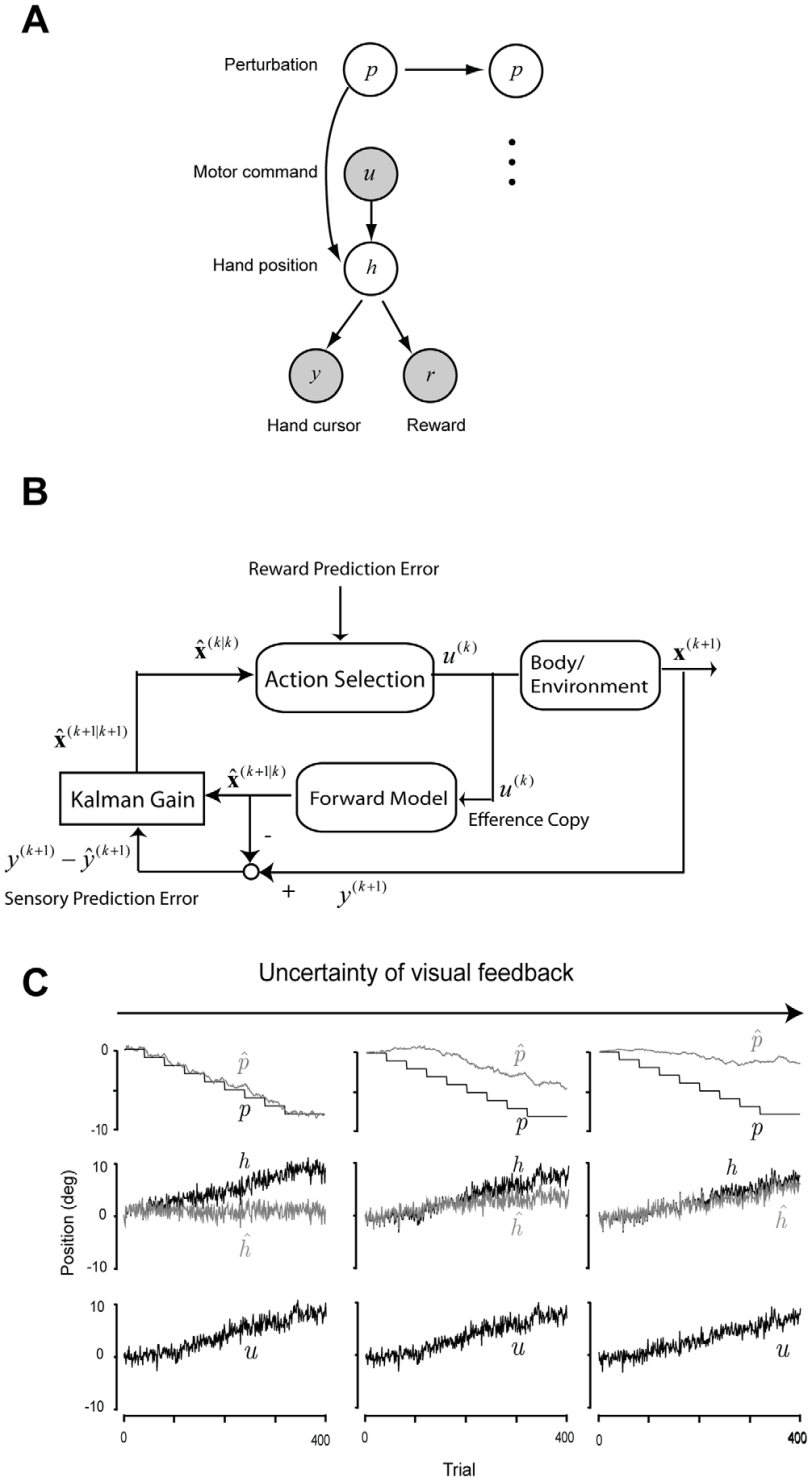
The theoretical problem of learning motor control. (**A**) A generative model of the motor adaptation task. Motor commands are corrupted by a perturbation, which result in a hand position that is sensed via a cursor, and may also result in reward. The objective of the learner is to find the motor commands that maximize reward. White circles are hidden variables and gray circles are observed variables. Arrows indicate conditional probabilities. (**B**) Model of optimal learner. The learning system is composed of two compensatory mechanisms: action selector and internal forward model. At the trial k, the action selector outputs the motor command 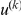 to make a transition of the state of the body and task from 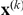 to 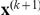. The state variable 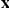 includes three elements: hand position *h*, perturbation *p*, and the position *t*. The brain observes the part of the state of the body 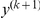. At the same time, the learner predicts the transition of the body state 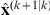 from the efference copy of the motor command. Kalman filtering correct the prediction to minimize the sensory prediction error 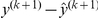 to have the updated state 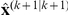. The action selector selects the optimal action as a function of the updated state at the next trial. (**C**) Sample disturbance and the response of the model. The task is to control the reach angle. Clockwise (CW) direction is positive and the target is at 0°. The uncertainty of the visual feedback was controlled to modulates the Kalman gain. The simulations predict a remapping regarding estimated hand position 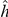 modulated by the level of visual uncertainty.

To explore the mechanism behind these findings, we considered a model of adaptation that relied on both sensory and reward prediction errors (Fig. 3A). Suppose that the brain generates a motor command *u*, resulting in a change in the state of the hand *h*, which also depends on a perturbation *p*. The nervous system senses the resulting motion of the limb *y* as well as whether that motion was rewarded *r*. Here, we considered a learner who updates motor command *u* to maximize reward. In theory, producing the motor commands that maximize probability of reward may rely on two kinds of learning: forming an optimal action selector, and forming an optimal state predictor (Fig. 3B). On trial *k*, action selector outputs motor commands 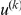. This depends on the estimated perturbation 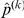 (which depends on sensory prediction error 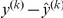), as well as the reward prediction error 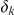. Therefore, in theory the trial-to-trial change in the motor commands is driven by two different error signals: the state estimator updated by the sensory prediction error, and the action selector updated by the reward prediction error.

An important prediction from this model is that reliance on the sensory prediction error is modulated by the Kalman gain, which is the ratio of estimation uncertainty to observation uncertainty. Therefore, if the uncertainty of visual feedback is large, the credit on the sensory prediction error becomes small, which makes the credit on the reward prediction error larger.

Fig. 3C shows results of simulations for different uncertainty levels of visual feedback. When the learner is provided with high quality visual feedback 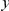 (analogous to ERR condition, Fig. 3C left column), it updates its estimate of perturbation 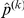, resulting in a motor-sensory remapping. As a result, the estimated hand position 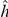 is near the location of the cursor and different from actual hand position 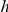. In contrast, when the learner is provided with uncertain visual feedback (analogous to EPE condition, middle column in Fig. 3C), the learner alters the motor commands using both the sensory prediction error and the reward prediction error. In this case, the adaptation produces a partial sensory remapping (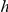 is not very different from 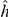 in the middle column of Fig. 3C). Finally, when the learner is provided with extremely poor visual feedback (analogous to RWD condition, right column of Fig. 3C), all that is available to the learner is success or failure (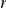  = 0 or 1). The learner still alters the motor commands to compensate for the perturbation, but the adaptation does not produce a sensory remapping (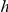 is not different from 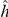 in the right column of Fig. 3C).

These three different patterns of sensory remapping generated by the model help explain the reason why we observed different patterns of sensory remapping in the three different paradigms. In the ERR condition in which high quality sensory feedback was available, adaptation produced large change in the state predictor, producing the sensory remapping. In RWD condition in which the visual feedback of the cursor was not available, adaptation focused on the action selector, which was updated by reward prediction error. Because this process did not involve a sensory remapping, we did not observe a change in the localization behavior of the subjects. In the EPE condition in which partial visual feedback was provided, learning depended on both an updating of the state predictor and the action selector. As a result, we observed the partial sensory remapping.

**Figure 4:**
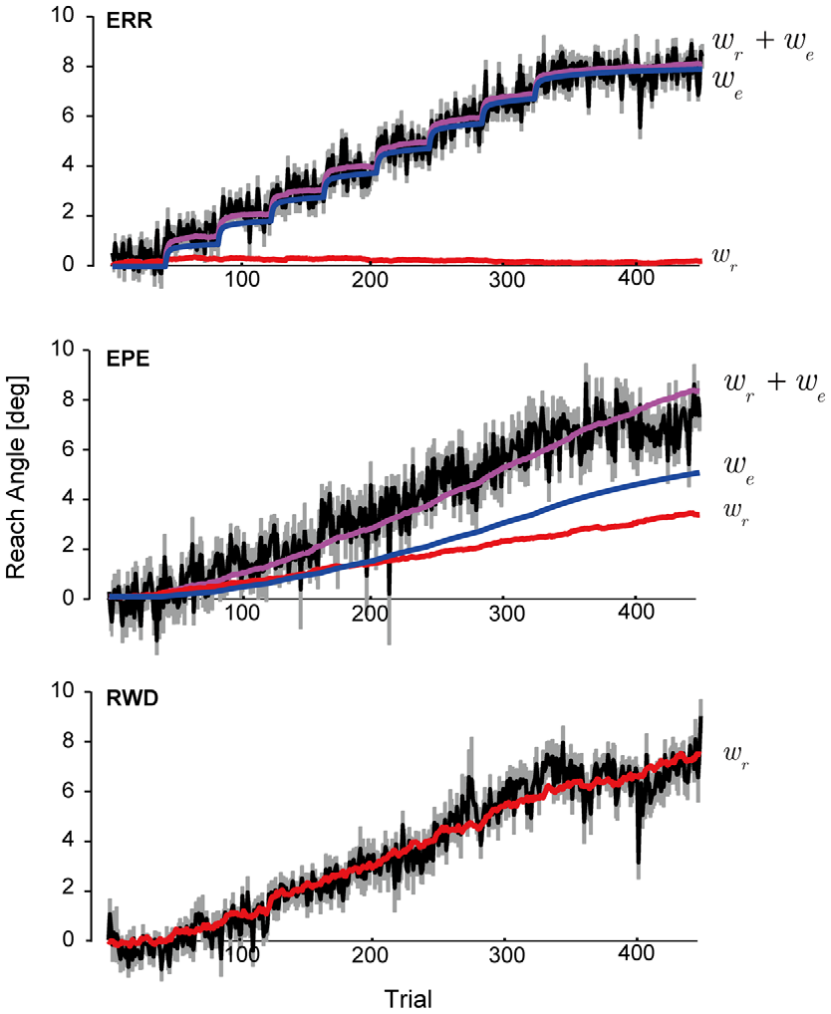
Estimated contribution of reward and sensory prediction errors to change in motor output during adaptation. When subjects experienced the ERR and EPE condition, we assumed that the motor commands were produced by the sum of two memories, 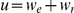, where 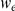 was updated by the sensory-prediction error and 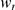 was updated by the reward prediction error. The best fit parameters predict the update of the two memories. The black think line is the averaged subject's reach angle during the adaptation period. The gray shadow is SEM. The superimposed purple line is the estimated reach angle from the model which is a combination of 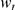 (red) and 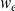 (blue). In the RWD condition, the motor commands are updated by only the reward-prediction error: 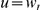.

To validate our model, we used it to estimate how much of the change in the motor commands that we observed in our subjects was due to each type of error. We imagined that the motor commands were generated by the sum of two states with a search noise, 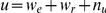, where 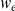 represents the estimate of the perturbation as updated by sensory prediction error and the 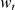 is updated by reward prediction error. Using a nonlinear optimization algorithm, we fit the model to the trial-to-trial behavior of each subject (reach direction on each trial), and the state of reward on that trial. In the RWD paradigm, the only feedback available was reward prediction error, i.e., 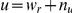. The results of our model fit are shown in Fig. 4 via the average of estimated parameters 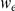 and 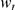, and their sum. These estimated values were superimposed on the average of subjects' trial-to-trial reach angle (black line) with SEM across subjects. In the ERR condition, by the end of adaptation the contributions of these two states were [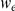, 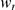] = [7.82,0.26]+/−[0.18,0.31]. Despite the fact that we used the exact the same model to fit the data for ERR and EPE, the best fit estimates of these two states in EPE were [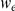, 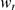] = [4.53,3.33]+/−[0.59,0.69], which were significantly different from those of ERR (ANOVA, F(1,18) = 18.93,p<0.001).

By fitting the model to the data, we were able to estimate the search noise 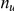. We found that the variance of the search noise in ERR was 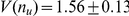, which was significantly smaller than that of EPE (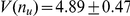, p<0.001), and RWD (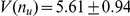, t-test, p<0.01). Our estimate of a significantly smaller search noise in the ERR condition is consistent with our inference that with high quality sensory feedback, the change in the motor commands is driven almost entirely by sensory prediction errors. This is also consistent with the fact that in the ERR condition, there was a scarcity of reward prediction error: In ERR, more than 95% of trials were rewarded, whereas the probability of reward in EPE was 83% and that in RWD was 76%. Therefore, our analysis suggests that in the ERR paradigm the change in the motor commands was due primarily to adaptation of the state estimator (accounting for the sensory remapping), whereas in the RWD paradigm the change was due to adaptation of the action selector (accounting for the lack of sensory remapping). In the EPE paradigm the change was due to both the state estimator and the action selector.

## Discussion

Our goal was to determine whether during motor adaptation one could dissociate between learning from reward prediction errors vs. learning from sensory prediction errors. We considered a reaching task in which visual feedback regarding cursor position was altered. The quality of this feedback was manipulated so that in one group the sensory feedback was of high quality (available throughout the reach, ERR group), in another group the sensory feedback was of low quality (available only at the end of the reach, EPE group), and in a third group the sensory feedback was unavailable (RWD group). All groups had access to reward (success or failure) at the end of their movement. We found that after a long period of training, all three groups adapted their motor commands. In the ERR group this adaptation accompanied a wide pattern of generalization and a significant change in the perceived sensory consequences of motor commands. In contrast, in the RWD group the adaptation accompanied a narrow pattern of generalization and no change in the perceived sensory consequences of motor commands. In the EPE group, generalization and sensory remapping were intermediate. Interestingly, in the EPE group individuals who demonstrated a larger sensory remap also had a wider generalization function. Increasing the uncertainty in the sensory prediction error altered both the width of generalization function and the amount of sensory remapping, while it did not affect the level of adaptation.

While previous models of motor adaptation have relied exclusively on sensory prediction errors to form an estimate of the perturbation [4], [5], [19], [20], the comparable levels of motor adaptation in our groups (ERR, RWD, and EPE) suggest that the brain relied on another source of error, the reward prediction error, when the sensory prediction error was not informative. In fact, it has been shown that the reward may modulate motor planning [21], [22]. Thus, it seems more rational that the purpose of learning is not merely to estimate the magnitude of a perturbation, but to produce motor commands that maximize reward [23].

We formulated this adaptation as a reward maximization process by assuming an “optimal learner”. The optimization relied on two update equations: one was the optimal estimator that inferred the state of the body, and the other was the optimal policy that selected the action as a function of the estimated state [24], [25], [26]. Based on this theory, our model of the optimal learner was composed of two components: reinforcement learning for action selection, and state estimation for identifying the sensory consequences of motor commands [27]. In this model, the objective of state estimation was to estimate the perturbation in the environment and the hand position as a consequence of the motor command, while the objective of the reinforcement learning was to update how to select the action to maximize reward probability [28]. The simulation showed that the learner relied mostly on the sensory prediction error in ERR paradigm. As a result, the learner updated the parameter associated with the sensory consequence of the motor command, which predicted the illusion that we observed in Experiment 1. In contrast with the ERR paradigm, the RWD paradigm did not provide the sensory prediction error. Thus, the simulation with the RWD paradigm showed that the reward-prediction error updated the action but did not change the estimate of hand position. Thus, high quality sensory feedback produced learning that depended primarily on sensory prediction errors.

While our model was not designed to account for the distinct generalization patterns in the ERR and the RWD paradigms, previous studies have speculated that generalization patterns are a reflection of the neural encoding of information during learning [16], [29]. For example, generalization patterns during reach adaptation in force fields appear consistent with an encoding in which the neurons have activity fields that resemble those in the primary motor cortex [13], [30]. In contrast, generalization patterns in visuomotor rotations appear more consistent with an encoding similar to cells in the posterior parietal cortex [31]. In this framework, the two different generalization patterns seen in RWD and ERR paradigms suggest engagement of two different neural mechanisms that each learn from reward and sensory prediction error. Another possibility, however, is that the two forms of prediction error converge on a single neural structure that guides motor learning.

By presenting the optimal learner model that includes two forms of prediction error, we built a connection between two disparate areas of research that has focused on different parts of the brain. Motor adaptation has focused on tasks that typically depend on the integrity of the cerebellum [32], [33]. Habit learning [34], visuomotor sequence learning [35], or action selection [36], [37] have focused on tasks that depend on the integrity of the basal ganglia [38], [39]. In fact, goal directed action in habitual learning is mediated by two representations: a representation of the instrumental contingency between the action and the outcome, and a representation of the outcome as a goal for the agent [40]. Because motor adaptation is also a goal directed action, the two learning mechanisms observed in this paper might be the general systems involved in a broad category of procedural learning. For example, these two distinct memories might be mediated by parallel cortico-basal ganglia mechanisms with different sensory domains [35].

Patients with basal ganglia disorders show little or no deficits in motor adaptation paradigms like force fields [33] or visuomotor perturbations [41], [42] (although patients with PD appear to show a deficit in consolidation of the memory [43]). Why is this? Our theory provides a potential answer: in the typical force field or visuomotor tasks, high quality sensory feedback is available, making it likely that sensory prediction errors play a dominant role. Because learning from sensory prediction errors likely depends on the integrity of the cerebellum [2], [32], [44], the implication is that the ability of basal ganglia patients to adapt to visuomotor and force field perturbations is not evidence for normal motor adaptation, but rather evidence for the idea that changes in motor output in these tasks are primarily driven by sensory prediction errors. The other implication of the theory is that the inability to adapt the sensory consequences of motor commands did not prevent adaptation of the motor commands in response to reward prediction errors. Indeed, when we altered the adaptation paradigm and made it so that changes in the motor output were driven by reward prediction errors, we found that in response to the reward prediction error subjects altered their motor commands. This theory predicts that by providing rewards appropriately during a motor adaptation task, the cerebellar patients may be able to update their motor commands without sensory recalibration.

Another implication of the theory is that the active search noise to explore the motor commands plays an important role in updating the action selector. Indeed, we found that trial-to-trial variability was modulated depending on types of error with significantly larger variability in the RWD than in ERR and in EPE. In previous studies, movement variability is generally thought to be due to signal dependent noise in the neuronal structures that generate motor commands [45], [46], [47]. However, noise is present even in the planning stage of movements [48]. Here, we found that during adaptation variability in movements was not due to meaningless noise, but an inherent part of a search that the brain engaged in to find motor commands that acquired a more rewarding state.

In summary, changes that take place in motor commands during adaptation are likely to be driven by both sensory and reward prediction errors. Learning from sensory prediction error alters the predicted sensory consequences of motor commands, leaving behind a sensory remapping. During motor adaptation, the reliance on reward prediction errors can be increased by degrading the quality of the sensory feedback. Learning from reward prediction error does not accompany a sensory remapping. It is likely that the neural basis of learning from sensory and reward prediction errors are distinct because they produce different generalization patterns.

## Methods

### Experimental Procedures

Subjects sat in front of a robotic arm and held its handle [25]. A video projector painted the screen that covered the manipulandum and the subject's arm. A trial began by the robot positioning the subject's hand in a start box, at which point a target of 6° width appeared at 10 cm. Subjects were instructed to perform a ‘shooting’ motion so that their hand crossed within the target area, at which point the target was animated to show an explosion, and a score was increased by one point. In the error-based learning (ERR) paradigm, the cursor position was displayed during the movement toward the target. In the reward-based learning (RWD) paradigm, the cursor position was not displayed. For both groups, target explosion indicated success of the trial. The cursor was not displayed during the return of the hand to the start position.

### Ethics Statement

Protocols were approved by the local IRB and all subjects signed a consent form.

#### Experiment 1: Learning from sensory prediction errors

Volunteers (n = 14, 26±4.7 years old) were assigned to the ERR (n = 7) or the RWD group (n = 7). After a familiarization session, the experiment was composed of a visuomotor adaptation phase and two localization phases (PRE and POST). In the localization phase (Fig. 1A), the subjects performed four shooting trials followed by one localization trial. For the first 4 trials, the cursor was visible for the ERR learning group but invisible for the RWD group. For the 5^th^ trial, the cursor was invisible for both groups. In the localization trial, neither the cursor nor the target was projected. In this trial, subjects pointed with their left hand (over the screen) to the estimated position of their right hand as it crossed the target area in the previous trial. That is, the subjects were asked to estimate the location of their right hand in the previous trial. These five trials (four shooting and one localization) were repeated 10 times for the PRE phase, and 10 times for the POST phase. The PRE localization phase was followed by an adaptation phase in which subjects experienced zero-rotation with 40 trials and then the perturbation increased by 1° every 40 trials until it reached 8° (Fig. 1C). The 8° perturbation lasted 80 trials. After a short break, subjects experienced 96 additional trials with the 8° perturbation and then were tested in the POST localization task.

#### Experiment 2: Generalization

The idea behind this experiment was to test whether adaptation in response to sensory prediction errors (ERR paradigm) vs. reward prediction errors (RWD paradigm) differed in their generalization patterns. Volunteers (n = 27, 24±4.4 years old) were assigned to the RWD (n = 18) or ERR groups (n = 9). Both groups were provided with a familiarization session. Subsequently the subjects experienced two baseline blocks composed of 80 trials. In the baseline block, the target position was selected randomly from [−30°, −20°, −10°, 0°, 10°, 20°, 30°] with respect to the trained target. The frequency of the center target (0°) was 32/80 trials and that of each peripheral target was 8/80. The objective of the peripheral targets was to test generalization. During these trials the cursor was not displayed and the target did not explode. For the center target, an explosion was provided for both groups but the cursor was displayed for only the ERR group. The baseline phase was followed by an adaptation phase. In the adaptation phase, the target appeared at only the center direction (0°) and the subjects experienced zero-rotation with 40 trials and then the perturbation shifted every 40 trials by −1° until it reached −8° and was held at this level for 80 trials. After a short break, subjects experienced another 3 blocks of 48 trials with −8° perturbation. Finally, we tested the generalization of this adaptation via a protocol that was the same as pre-adaptation.

#### Experiment 3: End point error paradigm

Volunteers (n = 11, 26.1±5.2 years old) were recruited for the end-point error group (EPE). The target was located at one of seven position [−30 −20 −10 0 +10 +20 +30] degree with respect to the center line, along a boundary circle with a 10 cm radius. At the moment the cursor passed through the boundary circle, the boundary pass point (endpoint) was marked by the cursor for 200 ms. After a familiarization block, the subjects experienced the baseline block for the generalization task which is the same as Experiment 2, followed by the PRE localization block which is the same as the Experiment 1. Then, subjects experienced the adaptation blocks which is the same as Experiment 1, where the perturbation was gradually increased up to −8 degree which was followed by another 2 blocks of 48 trials with −8° perturbation. Next, we tested the generalization of this adaptation via a protocol that was the same as pre-adaptation. Finally, the subjects experienced the POST phase of the localization task.

#### Data analysis

The endpoint of the movement was defined as the intersection between the hand path and a 10 cm radius circle centered at the start position. The reach angle was calculated as the angle between the center of the target and the line that connects the start location and the reach endpoint. With respect to the midline, a clockwise rotation was defined as positive.

#### An optimal learner

Let us cast the problem of adaptation in a framework in which the brain predicts the sensory and reward consequences of motor commands, and then learns from prediction errors in both modalities. Hand position 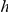 depends on the motor command 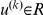 (initial reach direction) and is influenced by noise 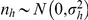:



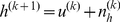
(1)


The units of all variables in Eq. (1) are degrees. The hand position controls the cursor position 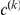, in which the perturbation 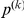 is imposed during the trial:



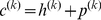
(2)


On trial *k*, subjects observe their hand position via a visual cursor at 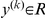 but cannot observe the perturbation directly:



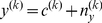
(3)where 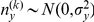 represents perceptual noise. Because subjects observe the hand position indirectly, we suppose that they predict hand position using the efference copy of the motor command



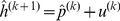
(4)where 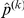 is the estimate of the perturbation. As subjects are repeatedly exposed to the perturbation, they build a prior knowledge of the characteristics of the perturbation: perturbations are correlated from trial to trial, and are also affected by noise 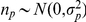 [4], [49], [50]:



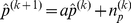
(5)


Set the extended state of the system as 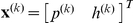. We then have a state update equation that relates motor commands with changes in state:



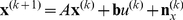
(6)where 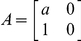, 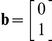, 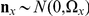, 
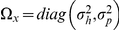
, and the observation equation is:



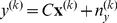
(7)where 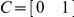. In summary, Eqs. (6) and (7) represent the relationship between motor commands and their sensory consequences. We assume that the objective for the learner is to maximize the rewards and minimize the cost. Under this assumption, for a linear dynamical system, optimal feedback control theory suggests that two interacting mechanisms are necessary: the optimal estimator and the optimal policy [24], [26]. The optimal estimator is composed of a forward model and a Kalman filter:



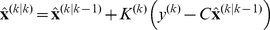
(8)where 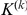 is the Kalman gain and 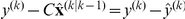 is the sensory prediction error. The Kalman gain is a function of the uncertainty of the estimated state and the measurement noise such that



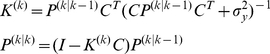
(9)where 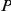 is the uncertainty of the state estimation and 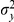 is the variance of the observation noise.

The optimal policy outputs motor commands as a function of the estimated state. In optimal control theory, the policy is computed from the end of the learning period backward [24]. However, in a learning problem, the learner updates the policy on every trial and the backward computation is not plausible. Thus, we used Actor-Critic architecture that enables it to find the optimal policy without backward computation [51]. Here we represent this policy with



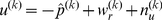
(10)where 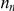 represents the active search noise to explore the motor commands and 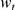 represents changes to the motor commands to maximize reward. Suppose that the expected cost-to-go function is of the form



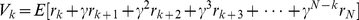
(11)for a general reward function 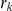 and discount rate of reward 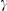. We used a standard temporal learning algorithm to solve this optimization problem [28], [51]. In this algorithm, the policy is updated to minimize the reward prediction error:



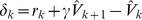
(12)where 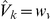. We used Temporal Difference (TD) error learning algorithm to updates the policy and the value.



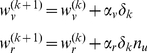
(13)


For our simulations (Fig. 3C), we used the following definition of the reward function:



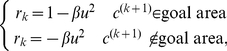
(14) where 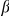 is the scaling parameter of the motor cost.

In summary, the learner has two kinds of prediction errors: a sensory prediction error (Eq. 8), and a reward prediction error (Eq. 13). The sensory prediction error updates an estimate of state produced by the motor commands (the sensory consequences of the action). The reward prediction error updates an estimate of the value of the states, and the policy that describes the ‘best’ motor commands to maximize reward.

#### Fitting the model to data

The previous section described how, in principle, one might alter the motor commands from trial to trial based on sensory and reward prediction errors. Here, we wished to fit this model to people's data and then test the predictions of the model. In the ERR and EPE paradigm, subjects were provided with both types of error, whereas in the RWD paradigm they were provided with only reward information. Our objective was to estimate contributions of each form of error to the change in motor commands during these three paradigms.

Our data from each subject consisted of the following: reach angle 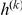, visual cursor 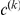 (both in units of degrees), and success or failure on that trial (reward) 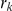. If a subject generated hand position 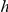 on a given trial, we assumed that this was related to three hidden variables: their estimate of perturbation 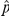, the accumulated change in the motor commands due to reward prediction errors 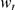, and an active search noise to find more rewarding motor commands 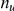:



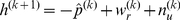
(15)


The problem is to estimate the variables of the right hand side from the measured sequence of hand positions. This requires solving an optimization problem. A rational cost is to minimize the squared difference between the observed sequence of hand positions and the sequence predicted by the model 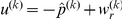. This is equivalent to minimizing the summation of magnitude of the active search noise 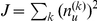. The constraint equations of this optimization process are Eqs. (8) and (13).

From Eq. (15) we have



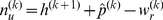
(16)where 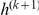 is the experimenter's observation of subject's hand position, 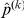
and 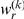 are the memory the optimal learner model updated. We will substitute Eq. (16) into Eq. (13) to update 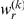 and 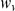.

We would also estimate the sensory prediction error is:



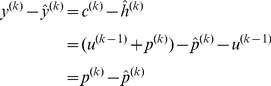
(17)where 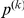 is the visual rotation that the experimenter imposed and 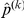 is the estimation of the perturbation that the optimal learner updated. Then, we substitute Eq. (17) into Eq. (8) in order to update 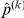.

Starting with initial conditions 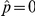, 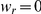, and 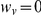, if we knew the unknown parameters [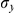, 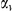, 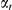], we could use the sensory prediction error in Eq. (8) to update 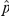, and the reward prediction error to update 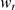 while updating the estimation of the value 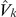 though updating 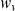. We searched for these three unknown parameters (using lsqnonlin in Matlab 6.5) in order to minimize the squared sum of difference between the model generated sequence of hand positions and the measured hand positions for each subject. We found that in the ERR paradigm, the average of the estimated parameters were [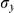, 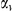, 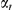] = [6, 0.15, 0.04] and in EPE paradigm, [69, 0.39, 0.03]. In the RWD paradigm, because no visual feedback of the hand position was provided, we assumed that motor commands were updated only by the reward prediction error. We set the Kalman gain to be zero and the average of the estimated parameters were [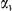, 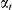] = [0.13, 0.14]. In the main document, we report the evolution of two memories and the sum of them: 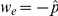, 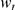, and 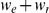.
